# Trigger Tool–Based Automated Adverse Event Detection in Electronic Health Records: Systematic Review

**DOI:** 10.2196/jmir.9901

**Published:** 2018-05-30

**Authors:** Sarah N Musy, Dietmar Ausserhofer, René Schwendimann, Hans Ulrich Rothen, Marie-Madlen Jeitziner, Anne WS Rutjes, Michael Simon

**Affiliations:** ^1^ Institute of Nursing Science University of Basel Basel Switzerland; ^2^ Nursing & Midwifery Research Unit Inselspital Bern University Hospital Bern Switzerland; ^3^ College for Health Care Professions Claudiana, Bolzano Italy; ^4^ University Hospital Basel Patient Safety Office Basel Switzerland; ^5^ Department of Intensive Care Medicine Inselspital Bern University Hospital Bern Switzerland; ^6^ Institute of Social and Preventive Medicine University of Bern Bern Switzerland; ^7^ Institute of Primary Health Care (BIHAM) University of Bern Bern Switzerland

**Keywords:** patient safety, electronic health records, patient harm, review, systematic

## Abstract

**Background:**

Adverse events in health care entail substantial burdens to health care systems, institutions, and patients. Retrospective trigger tools are often manually applied to detect AEs, although automated approaches using electronic health records may offer real-time adverse event detection, allowing timely corrective interventions.

**Objective:**

The aim of this systematic review was to describe current study methods and challenges regarding the use of automatic trigger tool-based adverse event detection methods in electronic health records. In addition, we aimed to appraise the applied studies’ designs and to synthesize estimates of adverse event prevalence and diagnostic test accuracy of automatic detection methods using manual trigger tool as a reference standard.

**Methods:**

PubMed, EMBASE, CINAHL, and the Cochrane Library were queried. We included observational studies, applying trigger tools in acute care settings, and excluded studies using nonhospital and outpatient settings. Eligible articles were divided into *diagnostic test accuracy studies* and *prevalence studies*. We derived the study prevalence and estimates for the positive predictive value. We assessed bias risks and applicability concerns using Quality Assessment tool for Diagnostic Accuracy Studies-2 (QUADAS-2) for diagnostic test accuracy studies and an in-house developed tool for prevalence studies.

**Results:**

A total of 11 studies met all criteria: 2 concerned *diagnostic test accuracy* and 9 *prevalence*. We judged several studies to be at high bias risks for their automated detection method, definition of outcomes, and type of statistical analyses. Across all the 11 studies, adverse event prevalence ranged from 0% to 17.9%, with a median of 0.8%. The positive predictive value of all triggers to detect adverse events ranged from 0% to 100% across studies, with a median of 40%. Some triggers had wide ranging positive predictive value values: (1) in 6 studies, hypoglycemia had a positive predictive value ranging from 15.8% to 60%; (2) in 5 studies, naloxone had a positive predictive value ranging from 20% to 91%; (3) in 4 studies, flumazenil had a positive predictive value ranging from 38.9% to 83.3%; and (4) in 4 studies, protamine had a positive predictive value ranging from 0% to 60%. We were unable to determine the adverse event prevalence, positive predictive value, preventability, and severity in 40.4%, 10.5%, 71.1%, and 68.4% of the studies, respectively. These studies did not report the overall number of records analyzed, triggers, or adverse events; or the studies did not conduct the analysis.

**Conclusions:**

We observed broad interstudy variation in reported adverse event prevalence and positive predictive value. The lack of sufficiently described methods led to difficulties regarding interpretation. To improve quality, we see the need for a set of recommendations to endorse optimal use of research designs and adequate reporting of future adverse event detection studies.

## Introduction

In recent decades, patient safety and quality of care have become a top priority in health care [1-3]. This has led to significant progress, especially regarding innovative use of electronic health records (EHRs). Adverse events (AEs), injuries attributed to medical care that are independent of the patient’s underlying condition, nevertheless remain a persistent problem. Apart from the impact on patients, they entail large human and financial burdens at every health care system level [4]. Regarding patient health, AEs’ negative consequences include extended hospital stays, higher readmission rates, and higher mortality [5]. Furthermore, AEs may lead to the patients’ and their families’ loss of trust in their health care professionals (HCPs), their health care system, or both [3]. The estimated prevalence of AEs in hospital inpatients ranges from 3% to 40% in acute care settings [2,6-10]. The wide range reflects the challenges involved in detecting and tracking AEs accurately [11].

To improve patient safety, health care organizations need valid and reliable tools to detect and assess AEs [12]. Several tools exist, but their ability to identify AEs is limited, and none of them are broadly accepted [13-15]. Currently, enumerating specific events that endanger patients depends mainly on voluntarily reporting by health care staff [16,17]. Systematic evaluations of this approach showed endemic underreporting, with only 2% to 8% of all harmful events being identified [18-20]. To depict the situation more robustly, the US Agency for Healthcare Research and Quality (AHRQ) published a set of Patient Safety Indicators (PSIs). Using administrative datasets, PSIs identify potential AEs, but are highly susceptible to variations in coding practice and are limited by many outcomes being easily concealed in the medical record [14]. Therefore, they miss a substantial fraction of AEs (low sensitivity), while producing a substantial fraction of false positive results (low specificity) [13,21].

One promising method is the Global Trigger Tool (GTT), developed by the Institute for Healthcare Improvement (IHI) [22]. Providing a structured method for identifying AEs from patient records [23,24], the GTT is a retrospective record review instrument that uses a list of triggers (or clues), ie, data elements within the health record, to alert reviewers to the potential presence of AEs [22,25]. By focusing on triggers within patient records, the GTT has demonstrated to identify up to ten times as many AEs as other detection methods [13]. Various studies have used the GTT, where some modified the methods, eg, by modifying the set of triggers, or by modifying the review process (eg, one reviewer instead of two for trigger identification). We refer to these modified versions as *trigger tool methodology,* reserving the term *GTT methodology* for the IHI’s original procedures [24].

The trigger tool was developed as a manual approach, ie, for application by HCPs reviewing patient records. Recently, an increasing interest developed for semi or fully automated AE detection methods using EHRs where lesser time and personnel resources are required for the AE identification [25-28]. *Prospective AE detection* would supply real-time feedback to HCPs, allowing timely interventions. The development of automated surveillance systems using EHR data has greatly facilitated AEs’ identification [28].

Semi or fully automated AEs detection methods show promise to efficiently measure AEs. Nevertheless, evidence need to be summarized based on the current literature to gather information for future development and implementation in a health care organization. As a variety of AEs’ detection methods exist, we decided to focus on trigger tool–based AEs detection methods only, allowing comparisons between studies as suggested in a previous systematic review on automated detection of patient harm [29]. As trigger tool methodology has shown higher sensitivity compared with other detection methods, we considered the manual trigger tool as the *gold standard*. This systematic review aimed to describe current study methods and challenges regarding the use of automatic trigger tool–based AE detection methods in EHRs in acute care settings. In addition, we aimed to appraise the applied studies’ designs and to synthesize estimates of AE prevalence and diagnostic test accuracy (DTA) of automatic detection methods using manual trigger tool as a reference standard.

## Methods

### Search Strategy and Study Selection

This systematic review followed the recommendations of the Cochrane Handbook for Systematic Reviews of Diagnostic Test Accuracy [30], the Cochrane Handbook for Systematic Reviews of Interventions [31], and the Preferred Reporting Items for Systematic Reviews and Meta-Analyses guidelines for the reporting of systematic reviews [32].

Hausner et al’s approach was applied to develop a robust search strategy (Multimedia Appendix 1) [33,34]. In PubMed’s basic search mode, we entered the following medical subject headings (MeSH) and free-text terms for title and abstract fields: (trigger OR triggers) AND (chart OR charts OR identif* OR record OR records) AND (adverse OR medical errors). The focus of the search was on “trigger” and not on GTT, as we aimed to include studies using variations of the original GTT. The search strategy was transposed to EMBASE, CINAHL, and the Cochrane Library, and terms were mapped to the appropriate keywords (eg, from MeSH to Emtree). Studies published in English, French, German, Italian, or Spanish were considered without restrictions concerning the years of the publication. In addition to searching the bibliographic databases, the team identified additional relevant literature from most common journals publishing in the field of trigger tool: *BMJ Quality & Safety*, *Journal of Patient Safety*, and *International Journal for Quality in Health*. For pragmatic reasons, the research team decided to limit the hand search of the most common journals to the years 2014 to 2017. The search was conducted in November 2015 with updates in April 2016 and July 2017.

We included observational studies that applied a trigger-based tool to detect AEs in EHRs in any acute care setting. We defined the target population of interest as patients hospitalized for at least 48 hours for any reason. The evaluated trigger tool approach (index test) had to involve either semi (ie, one part of the process still manual) or fully automatic identification of AEs [29]. Regarding DTA studies, we opted for a reference standard that produced a relatively low rate of missed AEs alongside an acceptable rate of false positive test results (events flagged as AEs that, upon examination, did not qualify as AEs). As shown by Classen et al (2011), manual trigger-based tools met our target criteria [13]. We excluded studies from nonhospital settings (eg, long-term care), outpatient clinics, or that concerned with nonprimary research (eg, systematic reviews or editorials).

The eligible articles were divided into two sets: (1) an automated trigger tool in comparison with a manual trigger tool method for AE detection, potentially enabling the evaluation of the trigger tool’s DTA (*diagnostic test accuracy studies*) and (2) application of an automated trigger tool without cross-verification with a manual trigger tool method, enabling us to synthesize the prevalence of AEs and the applied methods. We refer to the latter group as “*prevalence studies* ” throughout this paper.

### Data Extraction

Two main reviewers (SNM and MS) each screened half of the retrieved titles and abstracts for relevance according to the criteria outlined above. The other members of the research team each screened a quarter of the retrieved titles and abstracts, allowing double screening for all citations. Full-text screening was independently assessed by the main two reviewers, where disagreements were resolved by discussion, or by consulting the entire research team, if necessary.

Detailed study information was extracted into tables by SNM and a master student as part of her training. We used standardized piloted extraction sheets to tabulate variables related to design, sample population characteristics, applied trigger tool methodology, type and number of reviewers and triggers, and outcome data expressed as AE prevalence (overall and by AE type). To estimate DTA, we used 2x2 tables. Whenever possible, we derived the positive predictive value (PPV) of the triggers used. PPV is calculated by dividing the number of true positive triggers related to confirm AEs by the total number of positive triggers.

Unresolved disagreements or uncertainties between SNM and the master student were discussed and resolved in the research team, which included experienced systematic review and GTT methodologists, clinicians, and nurses, each with more than 10 years of experience in their specialty.

### Quality Assessment

We assessed the risk of bias and the concerns regarding applicability of all included studies. With respect to DTA, we assessed the quality of the included studies with the QUADAS-2 instrument, which we adapted for use as recommended by its authors [35]. The tool consists of four domains: (1) patient selection, (2) index test, (3) reference standard, and (4) flow and timing. Each domain contains signaling questions for risk of bias and concerns regarding applicability except the domain flow and timing. Each signaling question has three answer options: yes, no, and unclear. On the basis of the overall rating of the reviewers, an assessment can be made in each domain concerning bias and applicability. For example, one signaling question in the domain patient selection is, “Was a consecutive or random sample of patients enrolled?” All adaptations of the instrument are explained in Multimedia Appendix 2.

For prevalence studies, the research team generated a new tool based on the structure of the QUADAS-2 instrument to assess the risk of bias and concerns regarding applicability. The new tool consisted of six domains: patient selection, reviewer and algorithm selection, automatic detection method, outcomes, and flow and timing. All but outcomes and flow and timing included a section on risk of bias and concern regarding applicability, including signaling questions (Multimedia Appendix 3). The goal of this quality assessment was to verify the quality of the semi or fully automated trigger tool studies by focusing specifically on algorithm development and the basis for choosing each trigger.

Quality assessment process was conducted by one reviewer (SNM), and each research team member received at least one study to compare the results with SNM. Members did each task individually; then all results were discussed jointly by the multidisciplinary research team.

### Statistical Analyses

As we anticipated a paucity of evidence on DTA data, we deemed formal meta-analyses not feasible. For the total number of AEs and each type of AE, we present percentages with 95% CIs for prevalence, PPV, and rate of false negative test results. For the prevalence studies, we provided percentages for prevalence and PPV with 95% CIs, for AE overall and per type of trigger.

## Results

### Search Strategy and Study Selection

**Figure 1:**
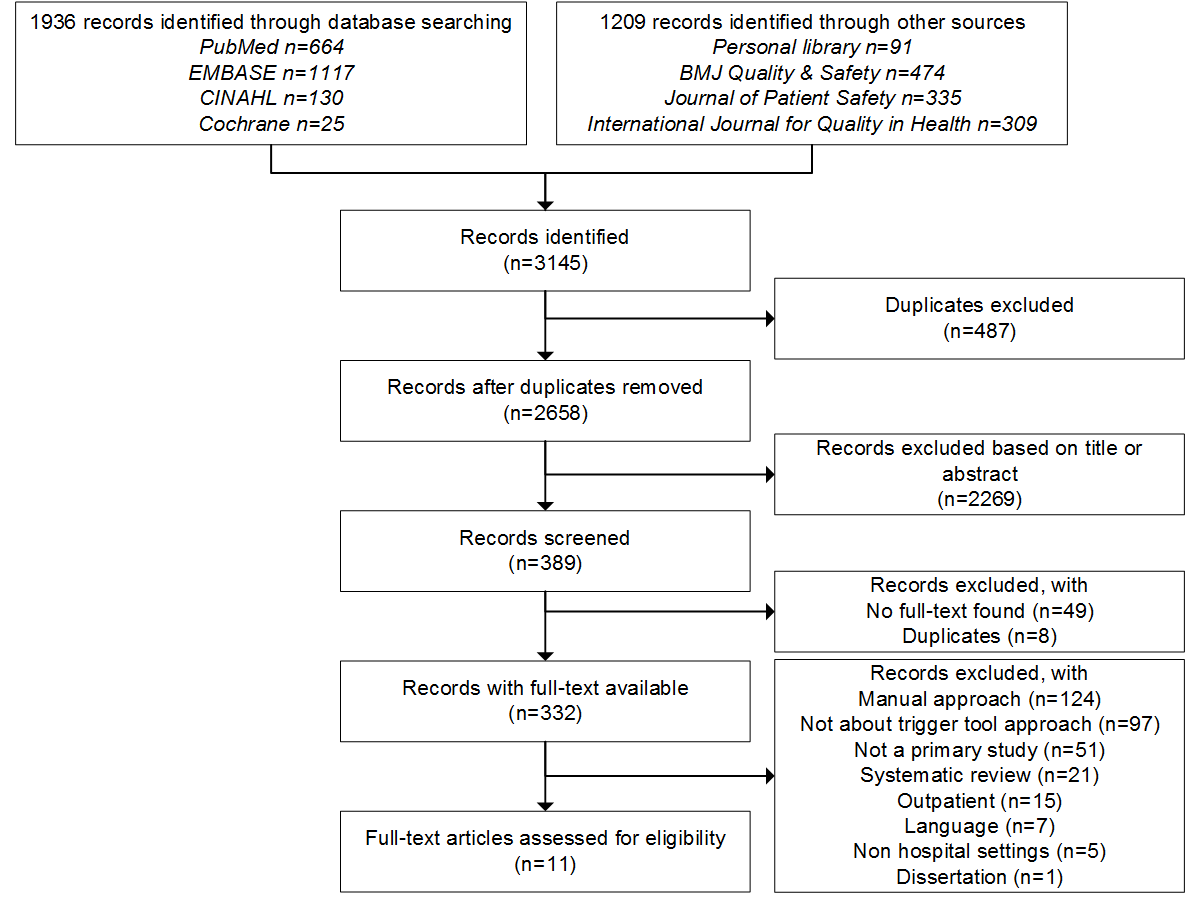
Flow diagram of the number of studies found with the search strategy, studies screened, and reasons for exclusions. Eleven studies fulfilled all inclusion and exclusion criteria.

After removing duplicates, 2658 citations were identified via our search strategy, the core journals, and our personal library. Of these, 11 met all selection criteria: 2 concerned diagnostic test accuracy studies and and 9 prevalence studies. A detailed view of the included studies is provided with a flow diagram in Figure 1.

### Description of Included Studies

The studies were published from 2005 to 2016. Of these, 9 were conducted in the United States [25,36-43], 1 in Denmark [44], and 1 in the United Kingdom [45]. Concerning the study populations, 7 were conducted among pediatric or neonatal patients [25,36,37,39,40,43,45], 3 among adults [38,41,42], and 1, published in abstract form only, provided no population information [44]. Two studies conducted multisite trigger tool research [25,42]. The bed capacity of the hospitals involved ranged from 26 to 1000 beds, with a median of 306. One study provided no information on bed capacity [36]. Further details concerning the design and the characteristics are summarized in Table 1.

One study explicitly followed the IHI guidelines for GTT [42], whereas the other studies used different trigger tool approaches or did not specify whether they followed the IHI guidelines. Concerning methodology, 6 studies addressed only adverse drug events (ADEs) [36,38,39,42,43,45], and only 1 used a fully automatic AE detection approach [44]. Definitions used to define and reference AEs varied [36-38,40-43,45] or were absent [25,39,44]. Seven studies used decision or agreement of the reviewer(s) to confirm an AE, without reference to the indexing method used [25,36-40,43]; and 2 studies omitted any explanation of the process [44,45]. Five studies did not consider preventability [38,39,42,44,45]; in the remainder, definitions varied. Three studies used the definition of a preventable AE as an “event leading to action deviating from the local standard of care” [37,40,43]; one study used a scale from “1—virtually no evidence for management causation” to “6—virtually certain evidence for management causation,” but without describing the applied cutoff [36]. One study used a 6-point confidence scale from “1—virtually no evidence of preventability” to “6—virtually certain evidence of preventability,” with a score ≥4 denoting preventability [41]; and one study merely described that preventability was determined by the reviewers’ decision [25]. Five studies assessed AEs’ severity using the National Coordinating Council for Medication Error Reporting and Prevention (NCC MERP) categories [25,37,39,40,43]; 4 studies did not state their assessment strategies [38,42,44,45]; one study categorized them according to four levels of severity (life-threatening, serious, clinically significant, or trivial) [41]; and one used NCC MERP categories together with the Common Terminology Criteria for Adverse Events version 4.03, ranging from mild (grade 1) to death (grade 5) [36]. Information concerning the data source, the triggers, and the reviewer(s) are detailed in Table 2.

Concerning the methodology, 5 studies came from the “Automated Adverse Event Detection Collaborative,” which is a consortium to facilitate the use of automated triggers in pediatric hospitals [25,36,37,39,40,43]. They all used the same approach, where an EHR-driven surveillance system was used, yet not providing detailed information on the software. Every night, trigger reports were automatically generated for laboratory results [37,40,43], medications levels in the blood [36,39], or both [25]. A clinical analyst examined every trigger by reviewing the EHR and interviewing care providers. The potential AEs were reviewed by specialists: pharmacists, physicians, endocrinologists, or anesthesiologists. The clinical analyst met with a multidisciplinary team, the “Automated Adverse Event Detection Steering Committee,” monthly to present the results. Two studies used natural language processing (NLP) to extract information from EHRs [41,44]. NLP is defined as a technique extracting information from narrative text and transforming it into structured text [41,46]. NLP is able to deal with synonyms, negations, and abbreviations used in narrative text. To build queries, SAS Text Miner tool [44] was used; however, no further details were provided. Structured Query Language [41] was used to identify AEs in the EHRs. The other studies used (1) Electronic trigger-detection messages that were sent automatically to two pharmacist reviewers [42], (2) Computerized trigger alert system that sent an alert to the project manager within 24 hours when conditions defined by the trigger algorithm were detected [38], and (3) electronic algorithms where triggers were identified automatically [45]. No further details concerning the development, the algorithms, or the tools used were given. Description of the methods are explained in Table 3.

**Table 1:** Design and characteristics of the sample and population of the included studies.

Study		Population	Time frame (months)	Sample size	Setting
**Diagnostic test accuracy studies**					
	Gerdes and Hardahl, 2013 [44]	Not stated	26	500	Not stated
	O’Leary et al, 2013 [41]	Adults, exclusion of patients admitted under observation status and cared for by either of the two medical record abstractors	12	250	General internal medicine
**Prevalence studies**					
	Call et al, 2014 [36]	Children	48	390	Oncology Hematology
	Dickermann et al, 2011 [37]	Children, exclusion weekend days for 5 months because of resource limitations	12	13,526	General internal medicine Surgical care Emergency department Intensive care unit (ICU) Psychiatric unit
	Lim et al, 2016 [42]	Adults	3x1 month	Not stated	Not stated
	Moore et al, 2009 [38]	Adults	5	456	General internal medicine Surgical care Obstetrics or gynecology
	Muething et al, 2010 [39]	Children	21 for one trigger and 16 for another one	Not stated	Not stated
	Nwulu et al, 2013 [45]	Not stated	12	54,244	Not stated
	Patregnani et al, 2015 [43]	Children	52 for one trigger; 40 and 60 for the others	Not stated	Pediatric ICU Neonatal ICU Cardiac ICU Medical and surgical acute care areas
	Shea et al, 2013 [40]	Children	36	6,872	Pediatric ICU Cardiac ICU
	Stockwell et al, 2013 [25]	Children, exclusion of emergency departments and ambulatory clinics	36 for hospital 1 and 51 for hospital 2	Not stated	General internal medicine Surgical care Psychiatric unit Neonatal Cardiac ICU Pediatric ICU

**Table 2:** Data sources, triggers, and reviewers of included studies.

Study		Data source	Triggers	Reviewer(s)
**Diagnostic test accuracy studies**				
	Gerdes and Hardahl, 2013 [44]	Unstructured and semistructured narrative texts in EHRs^a^	“Models,” not defined, identifying the most common triggers and/or AEs^b^	Not stated
	O’Leary et al, 2013 [41]	Enterprise Data Warehouse: EHRs or CPOES^c^; hospital and physician billing systems; incident reporting system; and admission or discharge or transfer with nightly updates from activities occurring in the preceding 24 h	Locally developed based on screening criteria from the Harvard Medical Practice Study and the IHI^d^ GTT^e^	Experienced hospitalists and physician-researcher (prior experience with the research method)
**Prevalence studies**				
	Call et al, 2014 [36]	EHR: laboratory, pharmacy, electronic medication administration record, CPOE, and documentation functions	Wide use in similar population and high likelihood to detect adverse drug events	Pharmacist and physician
	Dickermann et al, 2011 [37]	EHRs	Increasing use in hospitals’ protocols	CA^f^ trained
	Lim et al, 2016 [42]	EHR supports all inpatient and ambulatory care clinical and documentation activities	Review of literature and detectable in EHRs with reasonable PPV^g^	Pharmacists, medication safety pharmacist, and physician
	Moore et al, 2009 [38]	CPOE with decision support, EHR, clinical event monitors	Most common inpatient adverse drug events	Study investigators
	Muething et al, 2010 [39]	Clinical information system: computerized clinical order entry, clinical documentation, electronic medication administration record, data storage repository, and advanced clinical decision support	AEs steering committee	Endocrinologist, anesthesiologist, and frontline staff
	Nwulu et al, 2013 [45]	Locally developed electronic health and prescription computer system (laboratory results and prescribing, except some chemotherapy regimens) has built-in checks to identify potential prescribing errors (flagged through warnings and alerts)	Test the usefulness of two medication module triggers from the GTT proposed by IHI	Not stated
	Patregnani et al, 2015 [43]	EHRs	Clinical evidences	CA trained in the AE trigger process
	Shea et al, 2013 [40]	EHRs and Laboratory Information System	Clinical evidences and risks of deaths	CA trained in the AE trigger process
	Stockwell et al, 2013 [25]	EHRs	Multidisciplinary review process using several review criteria	CA

^a^EHRs: electronic health records.

^b^AE: adverse event.

^c^CPOES: computerized provider order entry system.

^d^IHI: Institute for Healthcare Improvement.

^e^GTT: Global Trigger Tool.

^f^CA: clinical analyst.

^g^PPV: positive predictive value.

**Table 3:** Overview of the automated trigger tool methodology.

Study		Description of the method
**Diagnostic test accuracy studies**		
	Gerdes and Hardahl, 2013 [44]	(1) Extraction and preparation of all texts from the EHRs^a^; (2) Use of SAS Text Miner and the SAS Enterprise Content Categorization software to build query models (natural language processing algorithms)
	O’Leary et al, 201 3[41]	(1) Leveraging of various information systems in the EDW^b^; (2) Write Structured Query Language queries to mimic work of a reviewer to identify potential AEs^c^ based on trigger tool; (3) Two reviewers review the positive EDW screens; (4) Another reviewer reviews narrative summaries and determines presence of AEs
**Prevalence studies**		
	Call et al, 2014 [36]	(1) Software program conducts an extensive search of patient records for any type of order containing specific medications and laboratory values; (2) Information generated into a report with patient-specific information; (3) Review by two reviewers
	Dickermann et al, 2011 [37]	(1) Trigger reports automatically generated on a daily basis from the EHR by querying the Sunquest Laboratory Information System for laboratory results; (2) Reviewer examined every trigger by reading the EHRs and interviewing care providers
	Lim et al, 2016 [42]	(1) Administration of a trigger drug to a patient automatically sent an electronic trigger-detection message to two reviewers; (2) Trigger-detection messages were evaluated immediately after or during the day by both reviewers (consensus if disagreement); (3) Event reviewed by a medication safety pharmacist and then by a physician for validation.
	Moore et al, 2009 [38]	(1) The laboratory results and administered medications of each adult hospital patient were continuously monitored by the computerized trigger alert system; (2) If any of the conditions defined was satisfied (trigger algorithm), an alert was triggered, and data were collected by study investigators on the patient for a period of 72 hours after the initial trigger firing to determine whether an adverse drug event had occurred.
	Muething et al, 2010 [39]	(1) Combination of trigger tool approach with the clinical information system; (2) Every evening, automatic detection of triggers are sent to the project manager (detection of event within 24 h); (3) Summary of the incident automatically generated and sent to the appropriate staff on the unit involved
	Nwulu et al, 2013 [45]	(1) The triggers identified electronically were linked to the electronic prescription records; (2) Two or more positive triggers generated for the same patient, within a 24- or 72-hour interval (trigger-dependent) were treated as one trigger; (3) The paper-based case notes were reviewed to identify any documentation of interest
	Patregnani et al, 2015 [43]	(1) Generation of a trigger report by querying the Laboratory Information System (2) Reviewer investigated the event by reading the patient’s EHRs and interviewing the clinical care team
	Shea et al, 2013 [40]	(1) Generation of a trigger report by querying the Laboratory Information System (2) Reviewer investigated the event by reading the patient’s EHRs and interviewing the clinical care team
	Stockwell et al, 2013 [25]	(1) Automated trigger reports are generated from hospital information systems on a nightly basis; (2) Each trigger report is examined by a reviewer and interviews conducted with care providers.

^a^EHRs: electronic health records.

^b^EDW: Enterprise Data Warehouse.

^c^AEs: adverse events.

Two studies compared results from automated trigger tool methodology with those obtained via the manual trigger tool method [41,44]. Six studies compared results with voluntary incident reports but did not cross-verify their results with those obtained via manually operated trigger-based tools; therefore, we considered these prevalence studies [25,36,37,39,43,45]. The remaining 3 studies did not compare their results with those obtained via any other method [38,40,42].

### Quality Assessment

**Figure 2:**
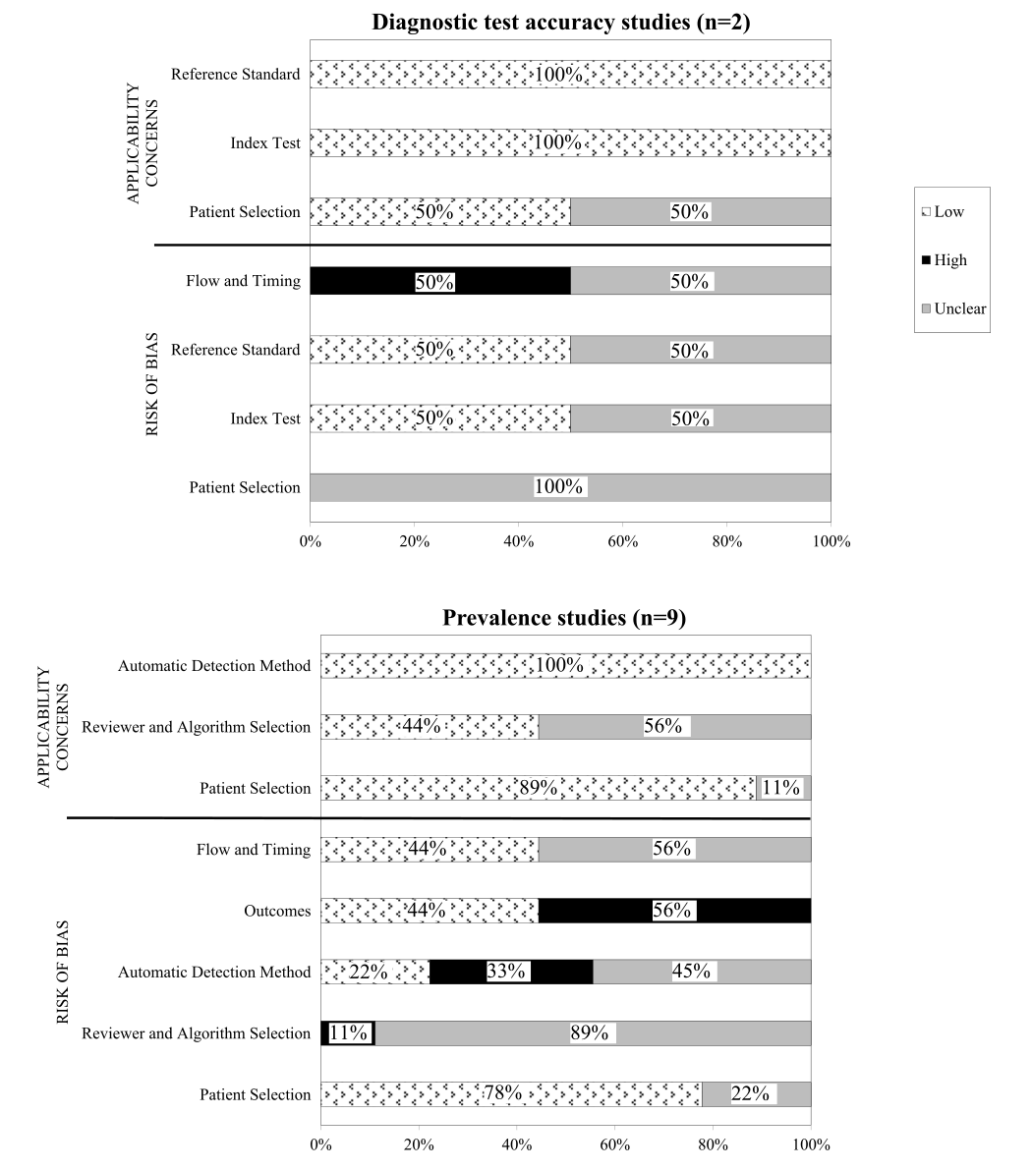
Risk of bias and concerns regarding applicability assessments for diagnostic test accuracy studies (upper panel) and prevalence studies (lower panel). Judgments are expressed as “low,” “high,” or “unclear” risk or concern for each of the domains (ie, “patient selection,” “index test”). The percentages refer to the percentage of studies meeting the judgment low, high, or unclear risk of bias or concerns regarding applicability in each of the domains. Quality Assessment tool for Diagnostic Accuracy Studies-2 (QUADAS-2) was used for the two diagnostic test accuracy studies and an in-house developed tool was used to assess the 9 prevalence studies.

Figure 2 shows detailed results of the two quality assessments.

#### Diagnostic Test Accuracy Studies

In one of the 2 studies, the assessment of all domains was hampered by poor reporting, and we were unable to judge the risk of bias and concerns regarding applicability [44]. In the other, we judged the concern regarding applicability of “patient selection” as low [41]. We judged a high risk of bias in the “flow and timing” domain and low bias risk and applicability concerns for the “index test” and “reference standard” domains [41].

#### Prevalence Studies

A total of 9 studies were selected as prevalence studies via our self-developed quality assessment tool (Multimedia Appendix 3). For “patient selection,” the bias risk was deemed low in seven studies [25,36-38,40,42,43] and applicability concerns were also low in eight of them [25,36-40,42,43]. We had no concerns regarding applicability of the automatic detection methods. As Figure 2 shows, for the other domain, we judged low bias risk and concerns regarding applicability in a minority of studies. In 5 studies, we judged a high risk of bias in the “outcomes” domain, mainly because their AE definitions did not reference those of the IHI or Food and Drug Administration, and they lacked clearly stated prevalence outcomes [37,38,42,43,45]. We judged high bias risks in 3 studies regarding the “automated detection method” domain [25,37,45]. In another, we judged the bias risk as high in the “reviewer or algorithm selection” domain [45] because the triggers were not consistently used or developed, and the reviewer lacked the required profile (eg, experience and training).

### Estimates of Diagnostic Test Accuracy, Prevalence, and Reliability

Estimates of DTA can be found in Table 4, with additional information concerning prevalence, preventability, and false negative rates for AE categories. No information concerning 2x2 tables were available for all the triggers; thus, the decision was made to use the AE categories.

**Table 4:** The table displays the estimates of diagnostic test accuracy in 2 studies comparing automated trigger-based tools with a manual trigger-based tool as reference standard.

Study	Type of adverse events	2x2 table for adverse events (True positive / false positive / false negative / true negative)	Prevalence^a^, % (95% CI)	Positive predictive value^b^, % (95% CI)	False negative rate^c^ (%)
Gerdes and Hardahl, 2013 [44]	Pressure ulcer	28 / 22 / 12 / 436	5.6 (3.6-7.6)	56 (42.2-69.8)	30
O’Leary et al, 2013 [41]	Adverse drug event	24 / 22 / 20 / N/A^d^	9.6 (5.9-13.3)	52.2 (37.7-66.6)	45.5
	Hospital acquired infection	7 / 11 / 4 / N/A	2.8 (0.8-4.9)	38.9 (16.4-61.4)	36.4
	Operative or procedural injury	5 / 4 / 4 / N/A	2 (0.3-3.7)	55.6 (23.1-88)	44.4
	Manifestation of poor glycemic control	3 / 2 / 5 / N/A	1.2 (−0.2 to 2.6)	60 (17.1-102.9)	62.5
	Pressure ulcer	0 / 8 / 2 / N/A	0 (0-0)	0 (0-0)	100
	Venous thromboembolism	5 / 1 / 0 / N/A	2 (0.3-3.7)	83.3 (53.5-113.2)	0
	Acute renal failure	2 / 1 / 0 / N/A	0.8 (−0.3 to 1.9)	66.7 (13.3-120)	0
	Delirium	0 / 0 / 0 / N/A	0 (0-0)	0 (0-0)	0
	Fall	0 / 0 / 0 / N/A	0 (0-0)	0 (0-0)	0
	Other	0 / 2 / 5 / N/A	0 (0-0)	0 (0-0)	100

^a^Prevalence is calculated by true positive/total number of patients.

^b^Calculated as triggers corresponding to an adverse event out of all triggers=true positive/(true positive+false positive).

^c^Calculated as false negative/(false negative+true positive).

^d^N/A: not applicable.

**Figure 3:**
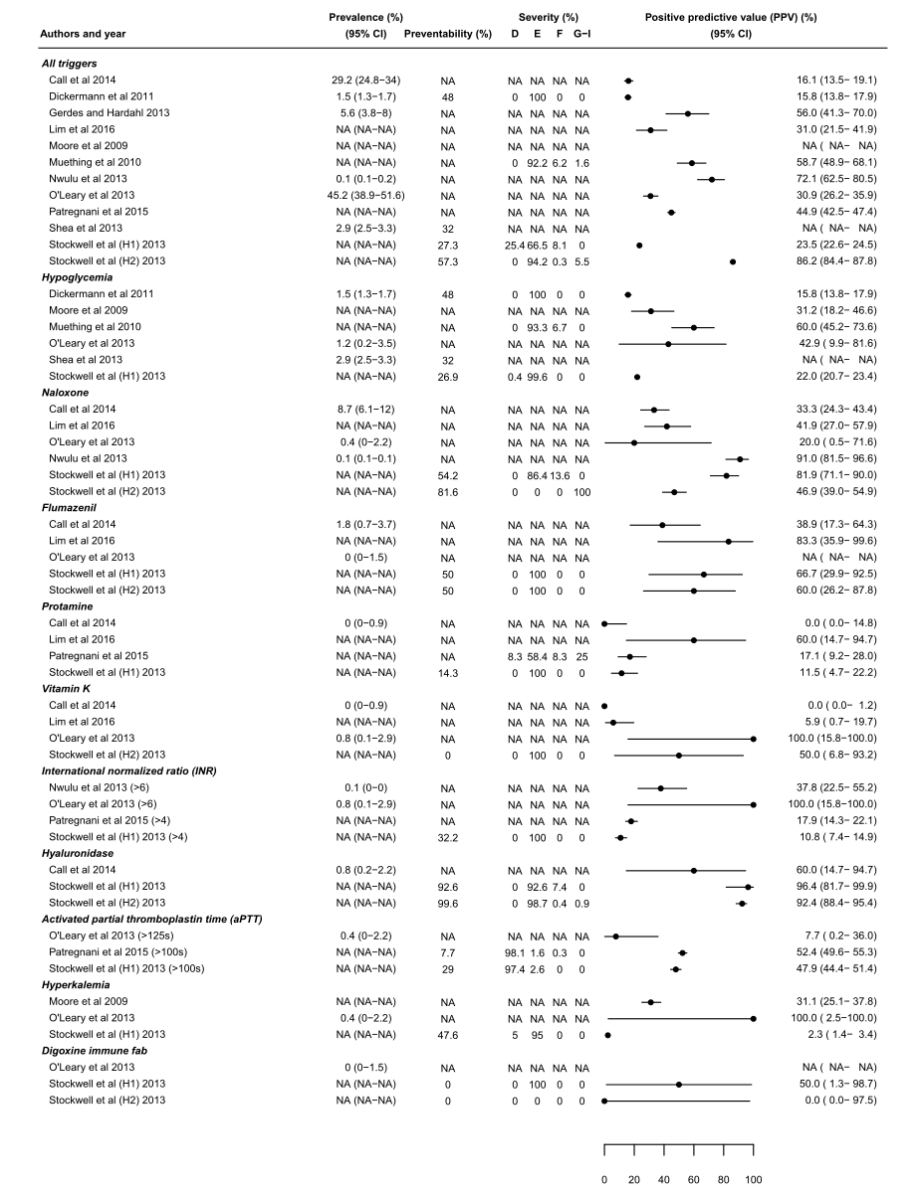
Prevalence, preventability, severity, and positive predictive value (PPV) for all the 11 studies. The figure begins with the results of all the triggers or adverse events (AEs) combined, then for each group of trigger order from the most studied to the least studied (part 1). Severity levels based on the National Coordinating Council for Medication Error Reporting and Prevention: D=an error that reached the patient and required monitoring or intervention to confirm that it resulted in no harm to the patient; E=temporary harm to the patient and required intervention; F=temporary harm to the patient and required initial or prolonged hospitalization; G=permanent patient harm; H=intervention required to sustain life; and I=patient death. H1: hospital 1; H2: hospital 2.

**Figure 4:**
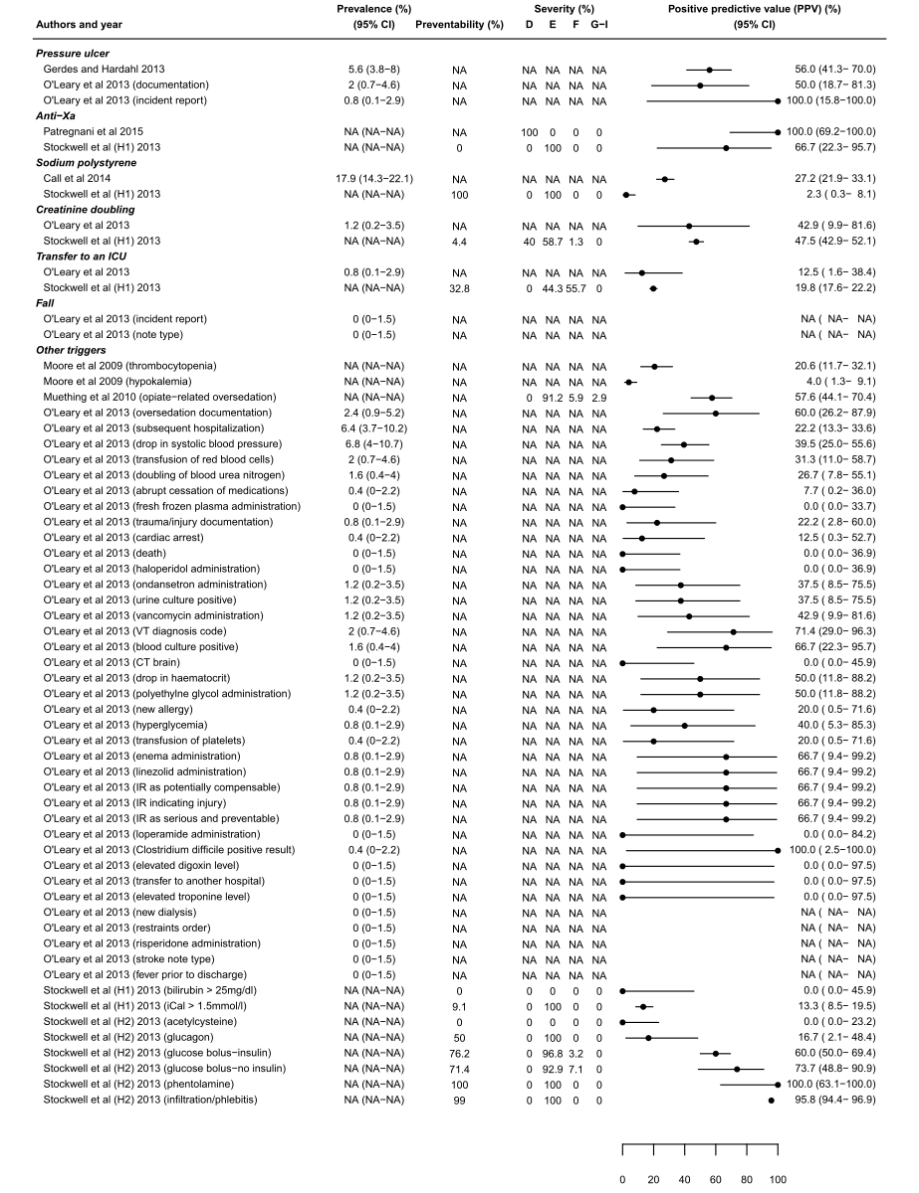
Prevalence, preventability, severity, and positive predictive value (PPV) for all the 11 studies. The figure begins with the results of all the triggers or adverse events (AEs) combined, then for each group of trigger order from the most studied to the least studied (part 2). Severity levels based on the National Coordinating Council for Medication Error Reporting and Prevention: D=an error that reached the patient and required monitoring or intervention to confirm that it resulted in no harm to the patient; E=temporary harm to the patient and required intervention; F=temporary harm to the patient and required initial or prolonged hospitalization; G=permanent patient harm; H=intervention required to sustain life; I=patient death. H1: hospital 1; H2: hospital 2; VT: venous thromboembolism; IR: incident report.

Across all the 11 studies, AE prevalence ranged from 0% to 17.9%, with a median of 0.8%. The PPV of all triggers to detect AEs ranged from 0% to 100% across studies, with a median of 40%. Some triggers are used in different studies and have different PPV values: (1) in 6 studies, hypoglycemia [25,37-41] had a PPV ranging from 15.8% to 60%; (2) in 5 studies, naloxone [25,36,41,42,45] had a PPV ranging from 20% to 91%; (3) in 4 studies, flumazenil [25,36,41,42] had a PPV ranging from 38.9% to 83.3%; and (4) in 4 studies, protamine [25,36,42,43] had a PPV ranging from 0% to 60%. We were unable to determine the AE prevalence, PPV, preventability, and severity in 40.4%, 10.5%, 71.1%, and 68.4% of the studies, respectively. These studies did not report the overall number of records analyzed, triggers, or AEs; or the studies did not conducted the analysis concerned. Detailed results for each trigger with prevalence, preventability, severity, and PPV are presented in Figures 3 and 4. Only 1 study supplied information on interrater reliability, reporting a kappa value of .52 to .78 [41].

### Challenges and Author Proposed Solutions

The challenges reported by authors concerned the relative large number of false alarms, the dependence of PPV on AE prevalence, and incomplete patient records leading to missed events. To reduce the fraction of false alarms, several authors suggested to use a threshold value for the triggers used based on patient characteristics [36,38,40,43]. For example, specific disease states of patients must have triggers with different threshold as the consequences might be stronger because of their disease. Authors from one study suggested to measure sensitivity and specificity instead of PPV, as latter is known to be largely affected by AE prevalence [38]. Another author group suggested to either improve completeness of patient information documentation or to combine different data sources to increase the chance to detect AEs as each data source contains different information type [42].

## Discussion

### Aim of This Review and Principal Findings

The goal of this systematic review was to synthesize the evidence concerning the development of a semi or fully automated method of AE detection in EHRs using trigger tools. The results show a broad variation in applied methods, selection of triggers, and estimates of AE prevalence and trigger-based PPVs. Insufficient reporting precluded full appreciation of the risk of bias and concerns regarding applicability.

Our findings are in line with another systematic review focusing on manual GTT [10]. Several interstudy differences can be hypothesized to explain the heterogeneity in the observed study estimates of AE prevalence and PPVs. These include the time frame (range: 1-51 months); the sample size (range: 250-54,244 records); the data sources and EHR system types; the automated approach; the review process; but also the differences in defining AEs, its severity, and preventability.

In addition, the choice and definition of triggers (eg, INR ≥6 [41,45] and INR >4 [25,43]) affect overall and per-trigger PPV. In some studies, only triggers identifying *unique* AEs are used for analysis, leading to varying results for similar triggers. Furthermore, the PPV is deemed to be strongly affected by the study’s AE prevalence. We could not test for this because of the reviewed studies’ heterogeneous definitions of prevalence. These included AEs per 100 patients admitted, AEs per 1000 patient days, or AEs per month. The missing information concerning the total number of patients included (36.4% of the 11 studies) hindered computation of prevalence estimates for these studies. The PPV of the triggers ranged from 0% to 100%. Even for the same trigger, high variability was observed.

Similar parallels can be observed regarding AEs’ severity. Even when naloxone was used as a trigger in two hospitals in the same study [25], although one hospital had a severity level of 96.4%, falling into category “temporary harm to the patient and required intervention,” the other had a severity level 100%, falling into the categories “permanent patient harm or patient death.” Several included studies suggested improvement of PPV values by limiting triggers to specific patient subpopulations, or by modifying thresholds based on age or other patient characteristics [36,40]. Moore et al [38] suggested using sensitivity and specificity instead of PPV, as the former is less affected by changes in AE incidence. The studies did not address difficulties or opportunities regarding the implementation of trigger tool–based methods, but we do not exclude that such information can be found in more qualitative types of research that we did not collect for this review.

The decision of several of the reviewed studies’ authors to employ semiautomatic approaches or to focus mainly on ADEs reflects the difficulty of detecting AEs fully automatically. The extracted high proportion of false alarms in various studies is likely explained in part by these difficulties. Although several studies showed that automatic tools can detect up to 69% of ADEs found manually, automatic detection of AEs overall remains problematic [47-50]. In our review, the sensitivity to detect AEs ranged from 0% to 100%. General AE detection requires more advanced technology, such as NLP, which can read and process free-text narrative [51,52], addressing complex issues such as negation and lexical variation of terminology. Previous research has suggested that automated AE detection methods were superior to manual tools [26,53,54]. Automated AE detection methods have the potential to screen large numbers of patients to save valuable time, which would not be possible by doing manually by human reviewers with the same accuracy. Yet, timely intervention is an important factor to avoid complications and patient harm when an AE is detected. Even if PPVs are not high for all AEs, automated AE detection methods provide an excellent alternative to the manual approach by saving time and resources [14]. As shown by the systematic review of Wang et al, the use of NLP with EHRs is still at its infancy, and closer collaboration of NLP experts and clinicians is missing [46]. Nevertheless, automated AE detection methods are a promising approach for patient safety improvement.

Looking deeper into the individual studies via our quality assessment tools, we realized that, for most, their methodology, their results, or both were reported in ways that were unclear, inconsistent, or incomplete, which challenged our risk of bias and applicability assessments. Several studies failed to report the number of records screened or the type of patients sampled. These elements, however, are crucial for interpretation of the various estimates and also for its reproducibility. In other studies, the number of triggers or AEs remained unclear. AE severity and preventability were not always reported, and the variation in use of definitions for AEs, severity, and preventability further hampered interpretation of estimates across studies. Interestingly, the majority of studies lacked to report PPV CIs, which is essential for the swift interpretation of the estimate’s precision.

Although not part of our quality assessment tools, we detected risk of selective outcome reprint in some studies. In these, severity and/or preventability assessments are reported in the Methods sections but not addressed in the Results section. Only one study checked for the presence of triggers at admission [42]. Such triggers or AEs should typically be excluded from the evaluation, as it cannot be targeted with interventions aiming at improving quality of care. Furthermore, although a reviewer’s expertise plays an important role in the detection of AEs, information concerning their professional background, experience, or training was mostly absent. Overall, there is substantial room for improvement of the quality of reporting.

### Limitations

Our decision to limit the inclusion criteria to studies concerning semi or fully automated trigger tool–like methodologies disqualified many studies, including those employing recommendations from the Harvard Medical Practice Study [28,55,56], machine learning [57], early warning systems [58-61], or other methods [62,63]. However, it allowed us to show that even within a narrow set of trigger-based tools, methods and outcomes varied considerably. The decision to exclude studies involving outpatients [62,64,65] or mixes of inpatients and outpatients [66] further decreased the number of eligible studies but increased the comparability of the patient population evaluated. Nevertheless, the overall low number of eligible studies precluded statistical evaluation of the impact of sources of variation and bias. The evaluation of diagnostic test accuracy is generally hampered by the absence of a widely accepted reference standard.

### Conclusions

This systematic review provides an overview about the application and outcomes of (semi) automatic trigger-based AE detection tools. We observed but could not formally explain the high degree of interstudy variation in reported estimates of prevalence and PPV, even in cases where similar triggers were tested. Although the AHRQ recently released common formats for event reporting [67], which supports the implementation of AE detection in the EHR, standards for the reporting of AE detection studies using trigger tools are lacking, yet urgently needed to overcome the methodological heterogeneity in future studies. We need better standards for reporting in this field of research to increase reproducibility, interpretation, and avoidance of research waste. A more standardized use of definitions of the types, severity, and preventability of AEs is desirable. We therefore call for a set of recommendations for the conduct and reporting of future studies and in the meantime, suggest authors, peer reviewers, and editors to pay special attention to complete reporting of study population, AE and trigger definitions, experience, training, and background of reviewers; methods employed to check for triggers and/or AEs at patient admission; and complete reporting of outcome data (numbers of triggers, nominators and denominators of the prevalence, and PPV).

## Appendix

Multimedia Appendix 1 Hausner et al’s approach.

Multimedia Appendix 2 Quality Assessment tool for Diagnostic Accuracy Studies–2 (QUADAS–2) instrument.

Multimedia Appendix 3 Trigger tool Quality Assessment Tool.

## Abbreviations

ADE: adverse drug event
AE: adverse event
AHRQ: Agency for Healthcare Research and Quality
DTA: diagnostic test accuracy
EHR: electronic health record
GTT: Global Trigger Tool
HCP: heath care provider
IHI: Institute for Healthcare Improvement
MeSH: medical subject headings
NCC MERP: National Coordinating Council for Medication Error Reporting and Prevention
NLP: natural language processing
PPV: positive predictive value
PSI: Patient Safety Indicator
QUADAS-2: Quality Assessment tool for Diagnostic Accuracy Studies-2
