# Supplementary material for: Trigger Tool–Based Automated Adverse Event Detection in Electronic Health Records: Systematic Review
Source: J Med Internet Res. 2018 May 30;20(5):e198. doi: 10.2196/jmir.9901 (PMC6000482; doi:10.2196/jmir.9901)
Supplement: Multimedia Appendix 1 [file jmir_v20i5e198_app1.pdf]

Multimedia Appendix: Hausner et al.'s approach

This is a Multimedia Appendix to a full manuscript published in the J Med Internet Res. For full copyright and citation information see http://dx.doi.org/10.2196/jmir.9901.

From our personal bibliographic archive and references of systematic reviews, we located 62 papers for the trigger tool methodology. We randomly divided those papers into two sets of 31: (1) Development set and; (2) Validation set. From the development set, we extracted the frequency of free text terms for title and abstract fields and subject headings (MeSH) with the PubMed PubReMiner tool (a free web service for searches in MEDLINE). This process identified potential candidate terms for inclusion in the search strategy. The search strategy's success was indicated by the number of results it returned and the sensitivity of the development and validation sets. The sensitivity is calculated by the number of papers found with the search strategy divided by the total number of papers in the set. For example, with the search strategy "trigger[tiab] OR triggers[tiab]", we found 31 papers out of 31 of the development set among the 99,429, meaning that it has a sensitivity of 100%. However, a high sensitivity is not enough: the number of results should be low to have a precise strategy. Sensitivities of the development and validation sets both should be around 90% with a low number of results. The table below shows the various steps with the final result (conducted on October 21st, 2015).

**Search Strategy**	**Results**	**Sensitivity development set**	**Sensitivity validation set**
trigger[tiab] OR triggers[tiab]	99,429	100% (31/31)	100% (31/31)
(tool[tiab] OR tools[tiab]) AND (trigger[tiab] OR triggers[tiab])	2,637	100% (31/31)	93.6% (29/31)
adverse[tiab] AND identif*[tiab]	50,525	80.7% (25/31)	87.1% (27/31)
adverse[tiab] AND (trigger[tiab] OR triggers[tiab] OR safety[tiab] OR medical errors[mh])	68,266	90.3% (28/31)	93.6% (29/31)
adverse[tiab] AND (trigger[tiab] OR triggers[tiab] OR detect*[tiab] OR medical errors[mh])	28,943	90.3% (28/31)	93.6% (29/31)
adverse[tiab] AND (trigger[tiab] OR triggers[tiab] OR record[tiab] OR records[tiab] OR medical errors[mh])	14,154	90.3% (28/31)	93.6% (29/31)
adverse[tiab] AND (trigger[tiab] OR triggers[tiab] OR chart[tiab] OR charts[tiab] OR medical errors[mh])	8,802	90.3% (28/31)	93.6% (29/31)
(adverse[tiab] OR medical errors[mh]) AND (detect*[tiab] OR trigger[tiab] OR triggers[tiab]) AND (chart[tiab] OR charts[tiab] OR record[tiab] OR records[tiab])	1,681	74.2% (23/31)	83.9% (26/31)
(adverse[tiab] OR trigger[tiab] OR triggers[tiab]) AND (detect*[tiab] OR identif*[tiab]) AND	6,157	87.1% (27/31)	93.6% (29/31)

(medical errors[mh] OR chart[tiab] OR charts[tiab] OR record[tiab] OR records[tiab])			
(trigger[tiab] OR triggers[tiab]) AND (adverse[tiab] OR record[tiab] OR records[tiab]) AND (chart[tiab] OR charts[tiab] OR medical errors[mh])	191	77.4% (24/31)	83.9% (26/31)
(trigger[tiab] OR triggers[tiab]) AND (chart[tiab] OR charts[tiab] OR identif*[tiab] OR record[tiab] OR records[tiab]) AND (adverse[tiab] OR medical errors[mh])	561	90.3% (28/31)	93.6% (29/31)
