# Supplementary material for: Trigger Tool–Based Automated Adverse Event Detection in Electronic Health Records: Systematic Review
Source: J Med Internet Res. 2018 May 30;20(5):e198. doi: 10.2196/jmir.9901 (PMC6000482; doi:10.2196/jmir.9901)
Supplement: Multimedia Appendix 2 [file jmir_v20i5e198_app2.pdf]

Multimedia Appendix: QUADAS-2

This is a Multimedia Appendix to a full manuscript published in the J Med Internet Res. For full copyright and citation information see http://dx.doi.org/10.2196/jmir.9901.

Phase 1: State the review question: To determine the sensitivity and specificity of the Global Trigger Tool in detecting adverse events in adult inpatients with an electronic/automatic approach, using the manual GTT application as the reference standard.

Patients (setting, intended use of index test, presentation, prior testing):<br>***All patients hospitalized for at least 48 hours (inpatient) with any specific disease***
Index test(s):<br>***Global Trigger Tool or a modified version (added/removed/modified triggers) used in a semi- or fully automatic way***
Comparator test:<br>***None***
Reference standard and target condition:<br>***Manual Global Trigger Tool or a modified version (added/removed/modified triggers)***

Flow diagram of the study

DOMAIN 1: PATIENT SELECTION

***A. Risk of bias**: Could the selection of patients have introduced bias?*

Describe methods of patient selection:

1. ***Was a consecutive or random sample of patients enrolled? Yes / No / Unclear*** *
   If all accessible patients were selected as a sample or if the process of sampling was done with the method of random sampling, the question will be answered as "yes".

2. ***Was a case-control design avoided? Yes / No / Unclear*** *
   We excluded case-control studies, as these were deemed inappropriate; therefore, this question will not be used.

3. ***Did the study avoid inappropriate exclusions? Yes / No / Unclear*** *
   This question will be answered with "no" if patients are excluded because of conditions known to be associated with false negative or false positive results. In these situations, automated trigger tool may fail (over/underdiagnose adverse events). Excluding such patient record forms may artificially inflate estimates of diagnostic test accuracy.

RISK: LOW / HIGH / UNCLEAR

***B. Applicability**: Are there concerns that the included patients and setting do not match the review question?*

Describe included patients (advanced vs early disease, symptoms, setting, prior testing, presence of alternative conditions, demographic features, intended use of the test, inpatient duration):

If a study did not meet the patient population as described in the objective there will be a high concern regarding its applicability.

CONCERN: LOW / HIGH / UNCLEAR

* *Refers to signaling questions of the original QUADAS-2 tool*
† *Refers to signaling questions added to the original QUADAS-2 tool*

DOMAIN 2: INDEX TEST

A. Risk of Bias*: Could the conducting or interpretation of the index test have introduced bias?*

Describe the index test and how it was conducted and interpreted:<br><br><br>*Description of the preventability*

1. ***Were the index test results interpreted without knowledge of the results of the reference standard? Yes / No / Unclear** **
   Less important for objective tests or if index test is interpreted prior to reference standard.

2. **Were the adverse events defined based on the U.S Food & Drug Administration (FDA) definition and/or on the Institute for Healthcare Improvement (IHI) definition?** ***Yes / No / Unclear*** †
   FDA definition: "*Adverse event means any untoward medical occurrence associated with the medical intervention(s) at the hospital, whether or not considered drug related.*" (https://www.accessdata.fda.gov/scripts/cdrh/cfdocs/cfcfr/CFRSearch.cfm?fr=312.32)

3. ***Was the assessment of severity of the adverse events based on the National Coordinating Council for Medication Error Reporting and Prevention (NCC MERP)? Yes / No / Unclear*** †
   – FDA definition: "Adverse event means any untoward medical occurrence associated with the medical intervention(s) at the hospital, whether or not considered drug related." (https://www.accessdata.fda.gov/scripts/cdrh/cfdocs/cfcfr/CFRSearch.cfm?fr=312.32)
   – IHI definition: "Any noxious or unintended event occurring in association with medical care." (Griffin FA, Resar RK. IHI Global Trigger Tool for measuring adverse events. Institute for Healthcare Improvement Innovation Series White Paper. 2009.)

4. ***Did the development of the algorithm involve clinician(s) and was it based on a test set or an empirical approach? Yes / No / Unclear*** †
   Involvement of a clinician is assumed/shown to improve validity as well as an empirical development approach (https://www.ncbi.nlm.nih.gov/pubmed/18487779; https://www.ncbi.nlm.nih.gov/pmc/articles/PMC2909812/).

RISK: LOW / HIGH / UNCLEAR

B. Applicability*: Are there concerns that the index test, its conducting, or its interpretation differ from the review question?*

* *Refers to signaling questions of the original QUADAS-2 tool*
† *Refers to signaling questions added to the original QUADAS-2 tool*

If test conducting, technology, setting or interpretation differ from your review question, the results may not be applicable. Example: a higher ultrasound transducer frequency has been shown to improve sensitivity for the evaluation of patients with abdominal trauma.

CONCERN: LOW / HIGH / UNCLEAR

* *Refers to signaling questions of the original QUADAS-2 tool*
† *Refers to signaling questions added to the original QUADAS-2 tool*

DOMAIN 3: REFERENCE STANDARD

***A. Risk of Bias**: Could the reference standard, its conduct, or its interpretation have introduced bias?*

Describe the reference standard and how it was conducted and interpreted:

1. ***Is the reference standard likely to correctly classify the target condition? Yes / No / Unclear** **
   We are aware that there is no globally accepted reference standard in this field but reason that the full manual chart review and the manual version of the GTT are at the moment the most sensitive methods to detect AEs, although we lack empirical data on the misclassification rates of the full manual chart review and the manual version of the GTT. In this situation, we judged yes if a full manual chart review or a manual version of the GTT were used, and no evidence was found in the study report that was suggestive of potential sources of bias related to the use/development of the full manual chart review and the manual version of the GTT.

2. ***Were the reference standard results interpreted without knowledge of the results of the index test? Yes / No / Unclear** **
   This question will be scored as "yes" if it is explicitly stated that the adverse event detection occurs without knowledge of the automatic GTT results. The reference standard should be interpreted blind to index test results. Related to degree of subjectivity and order of tests.

3. ***Is the inter-rater reliability clearly stated and sufficiently high? Yes / No / Unclear** †
   Although a clinical diagnostic test accuracy study should be conducted after reliability is more or less established, in the field of GTT, the evidence on reliability is variable. For this reason, we added a signalling question regarding reliability. We classified "yes" if the inter-rater reliability was assessed with sound methods, and was judged to be acceptably high.

RISK: LOW / HIGH / UNCLEAR

***B. Applicability**: Are there concerns that the target condition as defined by the reference standard does not match the question?*

Reference standard outcomes are decisive: if the reference standard does not detect the target condition defined in the review question, results may not be applicable. It is critical to choose a valid/optimal reference standard.

CONCERN: LOW / HIGH / UNCLEAR

* *Refers to signaling questions of the original QUADAS-2 tool*
† *Refers to signaling questions added to the original QUADAS-2 tool*

DOMAIN 4: FLOW AND TIMING

***A. Risk of Bias**: Could the patient flow have introduced bias?*

Describe any patients who did not receive the index test(s) and/or reference standard or who were excluded from the 2x2 table (refer to flow diagram):<br><br>*Describe the time interval and any interventions between index test(s) and reference standard:*

1. ***Was there an appropriate interval between index test and reference standard? Yes / No / Unclear*** *
   Delays between tests can cause misclassification due to recovery or progression to more advanced disease. The length of time which may cause such bias will vary between conditions.

   This item is not applicable to the comparison between automated versus full manual chart review or the manual version of the GTT, due to the retrospective nature of both tools. We typically scored yes, unless there was evidence that the time period verified by the manual and automated version of the tools differed. In cases where the latter was true, we judged "no."

2. ***Were all patients included in the analysis? Yes / No / Unclear*** *
   This question will be scored as "yes" if all patients who were recruited into the study were included in the analysis. No is scored if one or more patients are missing from the 2x2 table. As the study prevalence of AEs can be as low as 2%, we judged high risk of bias if 6% or more of the patients were missing from the 2x2 table–assuming that one-third of those missing would not be random, potentially affecting our estimates of the prevalence.

3. ***Was the completeness of electronic health records (EHRs) data discussed and addressed? Yes / No / Unclear*** †
   If yes, the risk of bias will be lower, since it takes into account missing data for the analysis.

RISK: LOW / HIGH / UNCLEAR

* *Refers to signaling questions of the original QUADAS-2 tool*
† *Refers to signaling questions added to the original QUADAS-2 tool*
