# Supplementary material for: Trigger Tool–Based Automated Adverse Event Detection in Electronic Health Records: Systematic Review
Source: J Med Internet Res. 2018 May 30;20(5):e198. doi: 10.2196/jmir.9901 (PMC6000482; doi:10.2196/jmir.9901)
Supplement: Multimedia Appendix 3 [file jmir_v20i5e198_app3.pdf]

Multimedia Appendix: Trigger tool Quality Assessment Tool

This is a Multimedia Appendix to a full manuscript published in the J Med Internet Res. For full copyright and citation information see http://dx.doi.org/10.2196/jmir.9901.

STATE your review question here, and answer all signalling questions and judgments in light of this review question.

1. To determine the prevalence of AEs as detected by an electronic/automated or semi-automatic trigger tool in various adult inpatient populations.
2. To describe the reliability of electronic/automated trigger tools
3. To explore methods of phenotyping if adverse event detection in EHRs was used in the international literature.

P Patient records of patients hospitalized at least 48 hours (inpatient) with any specific disease admitted to any ward

I Global Trigger Tool or a modified version (added/removed/modified triggers) used in an automatic or semi-automatic way

C Not applicable

O Prevalence overall, by type of AE, and by type of hospital or ward.

DOMAIN 1A: PATIENT SELECTION

Describe methods of patient selection:

***A. Risk of bias**: Could the selection of patient records have introduced bias?*

1. ***Was the participation rate of eligible persons at least 50%? Yes / No / Unclear***
   If the rate is less, this likely introduces bias (From http://www.nhlbi.nih.gov/health-pro/guidelines/in-develop/cardiovascular-risk-reduction/tools/cohort).

2. ***Was a consecutive or random sample of patient records enrolled? Yes / No / Unclear***
   Reflect if all the subjects selected or recruited were from the same or similar populations (including the same time period)? Were inclusion and exclusion criteria for being in the study pre-specified and applied uniformly to all participants in a consecutive manner? If all accessible patient records were selected as a sample or if the process of sampling was done with the method of random sampling, this question will be answered as "yes".

3. ***Did the study consider patients covering a broad range of indications for hospitalisation? Yes / No / Unclear***
   This question will be answered with "no" when patients with very different profiles are not considered by exclusion from study entry. Such exclusions are highly likely to alter the estimates of prevalence. This is a situation where GTT might over/underestimate adverse events. For example: exclusion of certain groups of patients due to extended lengths of stay or high numbers of transfers.

RISK: LOW / HIGH / UNCLEAR

***B. Applicability**: Are there concerns that the included patients and setting do not match the review question?*

If a study did not meet the patient population as described in the objective there will be a high concern regarding its applicability. In this specific review, we allow for a broad range of settings and study populations.

CONCERN: LOW / HIGH / UNCLEAR

DOMAIN 1B: REVIEWER/ALGORITHM SELECTION

Describe methods to sample patients to be included in the reliability estimations:<br><br>*State the determined number of reviewers:*<br><br>*State the number of patient records included in the reliability estimations:*<br><br>*State the number of replicated observations:*<br><br>*Describe the reviewer characteristics (e.g., training, experience):*<br><br>*Did raters judge/analyse the records independently (e.g., stage 1 screening)?*

A. Risk of bias*: Could the selection of reviewers/algorithms have introduced bias?*

1. **Was the selection of the reviewer(s) based on his/her experience and/or professional background in the clinical setting? *Yes / No / Unclear***
   A lack of experience on the part of the reviewer(s) in the clinical setting may introduce bias. For reviewers with appropriate clinical backgrounds the bias might be lower concerning misclassification rates.

2. **Were the reviewer(s) trained on using and applying trigger tool methodology? *Yes / No / Unclear***
   A lack of trigger tool training and application knowledge may introduce bias. For reviewer(s) with more training the bias might be lower.

3. **Do the reviewer(s) have experience in applying the trigger tool or another retrospective chart review methodology? *Yes / No / Unclear***
   A lack of trigger tool experience may introduce bias. For reviewer(s) with more trigger tool experience the bias might be lower.

4. **Did the study use a test and validation sets to develop the algorithm? *Yes / No / Unclear***
   The development of EHR algorithms always leads to choices (e.g., whether they should be geared towards sensitivity or precision). Using the split-half method is crucial to assess the efficacy of the proposed algorithm.

5. **Is the inter-rater reliability clearly stated and sufficiently high? *Yes / No / Unclear***
   Although a clinical diagnostic test accuracy study should be conducted after reliability is more or less established, in the field of GTT, the evidence on reliability is variable. For this reason, we added a signalling question regarding reliability. We classified “yes” if the inter-rater reliability was clearly assessed with sound methods, and was judged to be acceptably high.

RISK: LOW / HIGH / UNCLEAR

***B. Applicability**: Are there concerns that the reviewer(s) do not match the review question?*

For example, if the profile(s) of the reviewer(s) applying the trigger tool in the study differ substantially from those of healthcare professionals who would apply it in clinical practice, a high concern may arise.

CONCERN: LOW / HIGH / UNCLEAR

DOMAIN 2: AUTOMATIC DETECTION METHOD

Description of the method

A. Risk of Bias*: Could the conducting or interpretation of the index test have introduced bias?*

1. **Was the application of the algorithms fully automatic? *Yes / No / Unclear***
   Algorithms implemented in a semi-automatic way with different process steps are expected to introduce more bias (semi-automatic processes being more prone to errors).

2. **Did the development of the algorithm involve clinician(s) and was it based on a test set or an empirical approach? *Yes / No / Unclear***
   Involvement of a clinician is assumed/shown to improve validity as well as an empirical development approach (https://www.ncbi.nlm.nih.gov/pubmed/18487779; https://www.ncbi.nlm.nih.gov/pmc/articles/PMC2909812/).

3. **Was the selection of triggers based on literature review and/or consultation with a group of experts in the field? *Yes / No / Unclear***
   Empirical evidence or at least a biological rationale should exist for each of the triggers included in the tool. This particularly applies to *new triggers not part of the original IHI set of triggers.*

4. **Are the triggers used the same for all settings and/or the hospitals participating in the study? *Yes / No / Unclear***
   If differing versions of triggers are in use across participating units, a strong rationale should have been given to adapt them. For example, it is acceptable to adapt triggers according to national circumstances where a specific drug is not licensed.

5. **Was the presence of any adverse event checked/controlled at the admission of the patient to the unit/hospital? *Yes / No / Unclear***
   If not, the unit/hospital might conclude that those AEs were the consequences of their own care to the patients.

RISK: LOW / HIGH / UNCLEAR

B. Applicability

1. **Are there concerns that the trigger tool test, its conducting, or its interpretation differ from the review question?**
   If test conducting, technology, setting or interpretation differ from your review question, the results may not be applicable. For example: triggers are not related to the IHI GTT; AEs captured in the study are more triggers than AEs.

CONCERN: LOW / HIGH / UNCLEAR

DOMAIN 3: OUTCOMES

***A. Risk of Bias**: Could the definition of outcomes have introduced bias?*

Describe the definition(s) of AE:

Describe the how prevalence was measured:

Describe your judgment, if you deem the definitions to be standard, or deviating from our review definitions and describe if you deem reliability sufficiently high:

1. **Were the adverse events defined based on the U.S Food & Drug Administration (FDA) definition and/or on the Institute for Healthcare Improvement (IHI) definition? *Yes / No / Unclear***
   - FDA definition: "Adverse event means any untoward medical occurrence associated with the medical intervention(s) at the hospital, whether or not considered drug related." (https://www.accessdata.fda.gov/scripts/cdrh/cfdocs/cfcfr/CFRSearch.cfm?fr=312.32)
   - IHI definition: "Any noxious or unintended event occurring in association with medical care." (Griffin FA, Resar RK. IHI Global Trigger Tool for measuring adverse events. Institute for Healthcare Improvement Innovation Series White Paper. 2009.)

2. **Was the assessment of the adverse events' severity based on the National Coordinating Council for Medication Error Reporting and Prevention (NCC MERP)? *Yes / No / Unclear***
   Classification of the AEs' severity follow: http://www.nccmerp.org/sites/default/files/indexColor2001-06-12.pdf

3. **For prevalence, were the outcome measures (dependent variables) clearly defined? *Yes / No / Unclear***
   A lack of a clear outcome measure definition might lead to different results, thus introducing bias.

4. **For prevalence, were the definitions consistently applied across all study participants? *Yes / No / Unclear***
   If not, AE prevalence values might differ between patient groups, thus introducing bias.

RISK: LOW / HIGH / UNCLEAR

DOMAIN 4: FLOW AND TIMING

***A. Risk of Bias**: Could the patient flow have introduced bias?*

1. **Was the completeness of electronic health records (EHRs) data discussed and addressed? *Yes / No / Unclear***
   If yes, the risk of bias may be lower, since it takes into account missing data for the analysis.

2. **Were all patients included in the analysis of prevalence? *Yes / No / Unclear***
   This question will be scored as "yes" if all patients who were recruited into the study were included in the analysis. No is scored if one or more patients are missing. As the study prevalence of AEs can be as low as 2.x%, we judge high risk of bias if 10% or more of the patients are omitted from the AE prevalence calculations. This assumes that one third of the missing would not be at random, potentially affecting our prevalence estimates.

RISK: LOW / HIGH / UNCLEAR
